# Analyzing Gaze During Driving: Should Eye Tracking Be Used to Design Automotive Lighting Functions?

**DOI:** 10.3390/jemr18020013

**Published:** 2025-04-10

**Authors:** Korbinian Kunst, David Hoffmann, Anıl Erkan, Karina Lazarova, Tran Quoc Khanh

**Affiliations:** Laboratory of Adaptive Lighting Systems and Visual Processing, Technical University of Darmstadt, Hochschulstr. 4a, 64289 Darmstadt, Germanyerkan@lichttechnik.tu-darmstadt.de (A.E.); karina.lazarova@stud.tu-darmstadt.de (K.L.);

**Keywords:** eye tracking, gaze, traffic, horizontal fixations, vertical fixations, object detection, highways, country roads, urban roads, lighting design, automotive

## Abstract

In this work, an experiment was designed in which a defined route consisting of country roads, highways, and urban roads was driven by 20 subjects during the day and at night. The test vehicle was equipped with GPS and a camera, and the subject wore head-mounted eye-tracking glasses to record gaze. Gaze distributions for country roads, highways, urban roads, and specific urban roads were then calculated and compared. The day/night comparisons showed that the horizontal fixation distribution of the subjects was wider during the day than at night over the whole test distance. When the distributions were divided into urban roads, country roads, and motorways, the difference was also seen in each road environment. For the vertical distribution, no clear differences between day and night can be seen for country roads or urban roads. In the case of the highway, the vertical dispersion is significantly lower, so the gaze is more focused. On highways and urban roads there is a tendency for the gaze to be lowered. The differentiation between a residential road and a main road in the city made it clear that gaze behavior differs significantly depending on the urban area. For example, the residential road led to a broader gaze behavior, as the sides of the street were scanned much more often in order to detect potential hazards lurking between parked cars at an early stage. This paper highlights the contradictory results of eye-tracking research and shows that it is not advisable to define a holy grail of gaze distribution for all environments. Gaze is highly situational and context-dependent, and generalized gaze distributions should not be used to design lighting functions. The research highlights the importance of an adaptive light distribution that adapts to the traffic situation and the environment, always providing good visibility for the driver and allowing a natural gaze behavior.

## 1. Introduction

Headlamp light distribution optimization plays an ongoing important role in headlamp development. An understanding of the gazing behavior could give insight for optimal design of the headlamp light distribution of cars. Thus, the comparison of the gazing behavior between day and night, and between country roads, highways, and urban roads could provide information on how light distribution should be designed to ensure optimal visibility conditions at night. Gaze tracking during driving has long been an essential tool for studying the influence of light distributions on drivers’ gaze behavior and driving style. Given its significance, this paper begins with a comprehensive literature review. Building on these insights, a study design is then developed.

The focus of this literature review is on the observation of real-world studies in road traffic and the distribution of gaze of the test subjects on the road.

Road traffic is a highly complex situation and depends on an high number of parameters, including situation-specific parameters. Grüner et al. [1] attempted to characterize the various factors that influence gaze behavior; see Table 1.

They divided them into variable and non-variable environmental and organismic factors. However, as the purpose of this paper is to generate gaze distributions for different route segments, the literature review does not address variable factors. The non-variable environmental parameters, such as road geometry (curve, straight line), lighting conditions (day/night, street lighting, headlights), road type (multi-lane, pavement), and road environment (highway, urban, country), are the main focus here [1].

**Figure 1:**
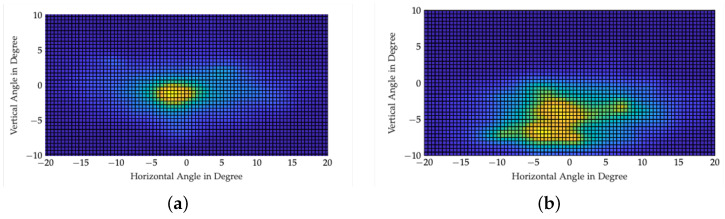
This figure shows (**a**) the fixation distribution during the day and (**b**) at night. The test subjects drove an approximately equally divided route consisting of city roads, country roads, and highways. The horizontal median ± std during the day was −0.86° ± 7.65° and the vertical median was vertical median ± std was −0.80° ± 3.1°. The horizontal median ± std at night was −0.82° ± 7.34° and the vertical median ± std was −2.04° ± 3.01°. The warmer the color (more yellowish), the denser the fixations in the plot [8].

When looking at the difference between day and night in gaze behavior in general, Graf & Krebs, Rockwell et al., Zwahlen, and Kobbert showed that the driver’s fixations are directed closer to the vehicle during nighttime drives than during the day [5,6,7,8]. This behavior was recently confirmed by Kobbert (2019) [8]; see Figure 1. For Kobbert, the vertical median ± std of gaze distribution was −0.8°±3.1° during the day and −2.04°±3.01° during the night. The overall accuracy and precision were reported to be below 2°.

At night, fixations appear to be limited to the illuminated area, while during the day the distribution of gaze is broader [9]. Brückmann also associated the narrower distribution at night with the light distribution of headlights [10].

A study by Cengiz et al. found that two out of three drivers’ gazes were higher on a lit country road than on the unlit test road during night driving. The most experienced driver did not show this. Nevertheless, this suggests an influence of the presence of road lighting on gaze behavior. Therefore, study design should consider differentiating between lit and unlit roads [11].

The number of fixations is higher at night than during the day [5,7,8]. The results of Cengiz et al. show that the gaze of an experienced driver and two inexperienced drivers differed between an unlit country road and a lit country road in that the gaze of the inexperienced drivers was raised on the lit sections [11]. No differences were found between day and night for the experienced driver.

Mortimer and Jorgeson investigated the influence of three different headlights: two low beams according to SAE and UNECE regulations, and a low beam that is symmetrical. On right-hand curves, the US headlamp and European headlamps with asymmetrical light distribution (higher luminous intensity on the right side) caused the fixation distribution to shift further to the right. This behavior was not observed for straight lines [12]. Damasky et al. showed that the targeted near-field illumination of halogen H4 lights leads to the gaze being directed closer to one’s own vehicle than with high-intensity discharge headlamps with an ellipsoid system and wide illumination close to the cutoff-line. A wider gaze distribution near the cutoff-line was considered here [13]. However, the data from Cengiz et al. showed that the location of most fixations did not correspond to the area of greatest illuminance [11].

All the different influences make it very complex to describe different situations and to determine a gaze distribution for several situations. Previous work has often made a distinction between urban streets, country roads, and highways.

A characteristic that is often not taken into account is the slope of the roads traveled. It is usually not apparent in the data where the actual vanishing point is located, as roads are not perfectly straight and even. For example, the data do not take into account whether the driver is driving into a valley or over a hill. Furthermore, the position of the vanishing point in the world camera image changes due to the vehicle’s pitch angle. Damasky and Huhn determined that a pitch angle of up to −1.8° occurs during braking, and +1.5° during acceleration in real driving [14,15]. Diem [16] stated that he had taken this into account in his work through video analyses.

### 1.1. Gaze in Urban Areas

**Figure 2:**
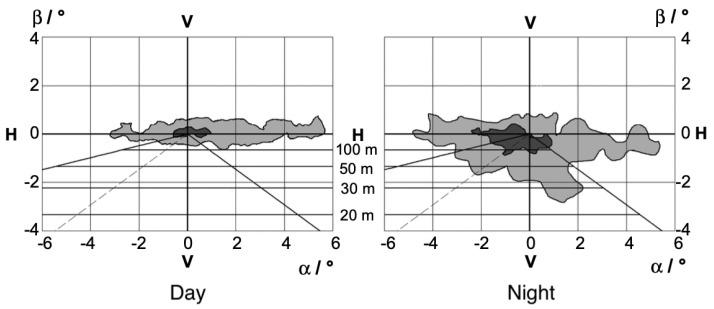
This figure shows the fixation distributions in the city during the day and at night. Here, α means the horizontal gaze distribution and β the vertical gaze distribution. The 10% range of the most frequent fixations is marked in dark gray and the 50% range in light gray [16]. In addition, visual ranges are drawn on the basis of a perfectly straight road.

Various studies show different results on how the road environment influences gaze distribution. Diem 2004 [16], for example, found that the distribution of gaze in the city during the day is relatively independent of the course of the road, but is largely determined by other road users such as parked cars, pedestrians or cyclists. More frequent and longer fixations occur at night. Diem explained this as the reduced peripheral perception at night. This results in the following fixation distributions in the city for day and night; see Figure 2.

Figure 2 shows that during the day, the median of the fixations tends to be centered and during the day the fixations tend to move to the left and there are closer fixations.

In general, the road environment of the city leads to a broader fixation distribution in the city than on country roads or highways [8,10,16].

The results of Kobbert [8] show that during the day the view is directed more to the left and above the horizon (day (median + std): horizontal −0.928° ± 9.982° and vertical: 0.604° ± 3.086°), and at night the view is clearly below the horizon and not as widely distributed as at night (night (median + std): horizontal: −0.834° ± 6.906° and median vertical: −0.811° ± 2.744°).

**Figure 3:**
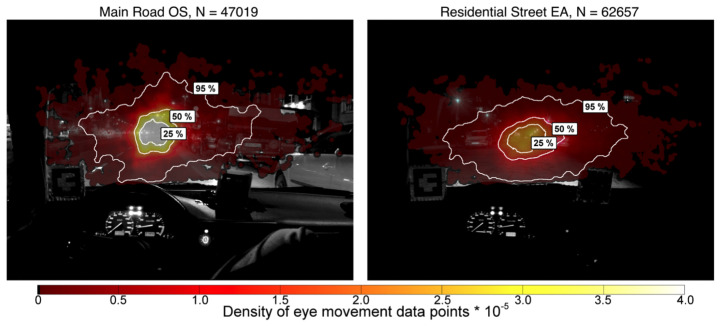
This figure shows the fixation distribution in the city at night on a main road (**left**) and a residential road (**right**) from the study by Winter et al. [17]. The 25%, 50%, and 95% areas of the most frequent fixations are shown. The density of fixations is also shown in color.

Winter et al. [17] considered a residential road with sodium vapor lighting, two lanes, parking on both sides, and intersections where driving on the right is prohibited, and a main road with high-pressure mercury vapor lamps, four lanes, and traffic lights. The researchers found that in the main road 95% of all fixations are best fitted by a 10° V/20° H (vertical, horizontal) ellipse, a two-standard-deviation (2SD) ellipse, or with a 10° circle around the main fixation locations, whereby the 10° circle was proposed to perform calculations for the adaptation luminance. In the residential area, the ellipse 10° V/20° H or 2SD° V/2SD° H was proposed to estimate the 95% area of all fixations; see Figure 3.

To summarize, the distribution of fixations is wider in the residential road than in the main road, and fixations tend to be directed to the right in order to quickly recognize or observe potential dangers such as pedestrians or cyclists. Intersections, with a “right-before-left” rule (the driver is inclined to look to the right in order to obtain information about whether driving is permitted) play a considerable role as well [17].

### 1.2. Gaze on Country Roads

On straight country roads, the data from Diem (2004) [16] show that during the day, fixations tended to be to the right and below the horizon, at a viewing distance of 120 m (on average, horizontal: 1.7° vertical: 120 m mean viewing distance (−0.6259° at 1.3 m camera height)). At night, the fixations are more centered and the gaze more lowered than during the day. Shinar et al (1977) [18] confirmed that the gaze is orientated more to the right during the day, but is slightly above the horizon (mean value: horizontal +1.6° vertical: +0.7°). Land and Lee (1994) [19], on the other hand, observed that fixations tend to be to the left and below the horizon. Schulz describes the gaze behavior on straight country roads during the day as follows: “The gaze moves around between the close-up area in front of the vehicle, the vanishing point of the road and objects at the roadside” [20].

Kobbert described the behavior on country roads in general, not just on straight sections. The data showed that during the day the fixations tend to be directed to the left and lie below the horizon, and at night the gaze wanders a little further to the left and the median lies above the horizon (day (median + std): horizontal: −0.768° ± 8.669°, vertical: −3.288° ± 2.733°; night (median + std): horizontal: −1.022° ± 6.594°, vertical: 0.742° ± 2.698°).

### 1.3. Gaze on Highways

On highways, according to Diem [16], three accumulation points occur on a two-lane highway during the day:

- In the left-hand lane (to keep an eye on overtaking traffic);
- On your own lane of traffic;
- On the emergency lane, or in the area of the traffic signs.

**Figure 4:**
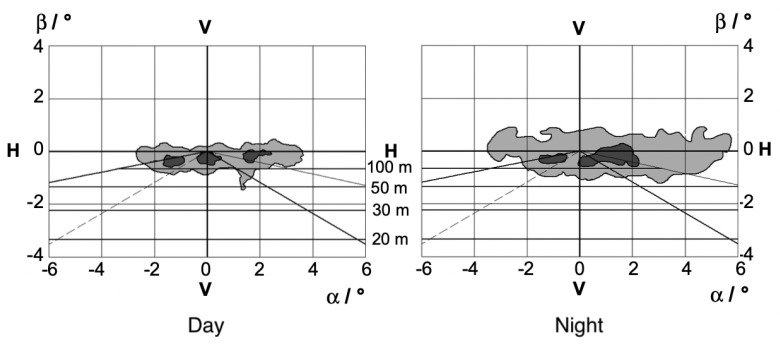
This figure shows the fixation distribution during the day and at night on a two-lane highway. Here, α shows the horizontal gaze distribution and β the vertical gaze distribution. The 10% range of the most frequent fixations is shown in dark gray and the 50% range in light gray [16]. In addition, visual ranges are shown on the basis of a perfectly straight road.

At night, on the other hand, the right and center lanes mix; see Figure 4.

The right fixations are slightly higher than the left fixations, which could be due to traffic signs or high trucks [16]. Hristov (2009) investigated gaze behavior exclusively on highways during the day. On a straight section of highway, he found that fixations were evenly distributed to the right and left and tended to be directed towards the sky. On average, however, fixations tended to be directed to the right (horizontal mean: +1°) [21].

The distribution of views on highways in general over different sections of the road, consisting of curves, straight sections, and two to four lanes, showed that fixations during the day tend to be on the left very close to the vanishing point or horizon of the road. At night, these shift slightly further to the left and slightly further upwards (day (median + std): horizontal −0.729° ± 7.647° and vertical −0.311° ± 2.987°; night: horizontal median −2.308° ± 5.775° and vertical −0.182° ± 2.896°) [8].

According to Diem, no difference can be recognized at night with dense traffic compared to daytime ([16]). At night, there is a mixing of the two right areas. The right fixation areas are slightly higher than those on the left. He attributes this to the position of traffic signs and high lorries [16].

The current study situation is not sufficient because the studies were often conducted with few subjects or a very long time ago (>50 years). Today’s eye tracking systems are more accurate than their predecessors due to technological advances. In addition, it is not recommended to make accurate statements about where drivers in traffic look on average in different situations without specifying the accuracy, without specifying how the system was calibrated. If the calibration choreography does not match the real conditions of the experiment, this can lead to errors. In this work, an approach is developed that allows the evaluation of eye tracking according to all non-variable environmental factors according to Grüner [1].

### 1.4. Lighting Distributions Based on Gaze Distribution

Kobbert et al. used the gathered Eye Tracking data, the object distributions gathered during the test drives, and an ideal light distribution based on detection studies to design a lighting distribution. In this study, it was suggested that areas containing a high concentration of “important” traffic elements, such as cars, pedestrians, or cyclists, should receive a significant amount of “attention”, which in this context is represented by fixations. Conversely, areas without such objects do not require the driver’s fixations [22,23,24].

**Figure 5:**
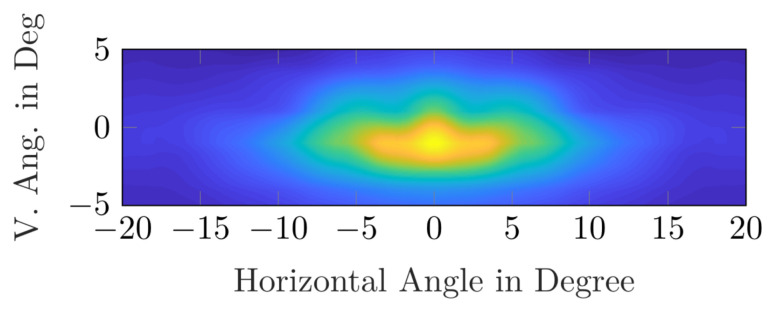
Mirrored and smoothed proposed new light distribution based on real traffic and gaze data as well as on detection tests on ideal road conditions according to Kobbert et al. [24]. The warmer the color (more yellowish), the denser the fixations in the plot.

They created the light distribution by subtracting the object distribution from the fixation distribution and multiplying the result by an ideal base light distribution. The resulting light distribution (see Figure 5) was designed to help the drivers see important objects that had not previously received “attention”.

### 1.5. Research Question

The literature review highlighted various approaches to investigating gaze behavior, as well as methods for designing automotive lighting distributions based on these findings. Building upon this foundation, this paper not only analyzes gaze behavior but also explores the following fundamental question:

**“Should gaze distributions be used to design headlamp light distributions for road illumination?”**

## 2. Materials and Methods

This section deals with the study design for the gaze analyses in road traffic. It was previously discussed in Section 1 that gaze behavior in real road traffic is dependent on multiple, highly intertwined influences. Non-variable environmental factors, such as the road geometry and surroundings, and variable environmental factors, such as other road users, signs, and displays, meet non-variable organismic factors of the test subjects, such as mental performance and variable organismic factors, such as fatigue or alertness.

It is, therefore, necessary to design a study that has measuring instruments to record all influences or to exclude specific influences in order to be able to make precise statements.

### 2.1. Measurement Setup

**Figure 6:**
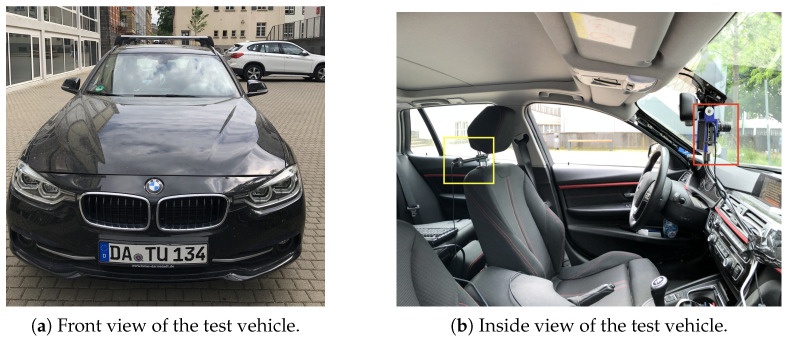
This figure shows in (**a**) the front view of the test vehicle. It is a BMW 3 Series Touring from 2016. Panel (**b**) shows the interior view with a view of the driver’s seat. The X1 illuminance measuring detector from Gigahertz-Optik is installed to the right of the driver’s seat (yellow). The Raspberry Pi, including the Raspberry Pi HQ camera, is mounted behind the rear-view mirror using a customized bracket (red). The GPS is on the roof.

The test vehicle used during the experiments was a 2016 BMW 3 Series, as shown in Figure 6. The vehicle was equipped with full LED headlights and did not have an adaptive front lighting system (AFS). The vehicle is the same vehicle used by Kobbert (2019) [8].

####  **Eye Tracking System** 

To provide gaze recording, while driving, the head-mounted eye tracker “Pupil Labs Core” [25,26] was used. It had the following configuration:

- World camera (moves with the head): 1280 × 720 px, @30 fps.
- 2 eye cameras 400 × 400 px @120 fps.
- Dark pupil measurement.
- 3D eye model with convergence calculation.
- Slippage compensation by the eye model.

The footage was taken with the “Pupil Capture” software v3.5, which was also used for calibration and validation of the system. Apart from the visual data, data of the GPS position of the subject and the illuminance levels of the environment were collected. Unlike previous studies, this research does not rely on a single accuracy value but, instead, accounts for gaze-angle-dependent accuracy for each participant. During validation, gaze angles were recorded and validated for each marker, allowing the creation of an individualized accuracy map for every participant. This approach ensures a more precise and context-aware representation of gaze accuracy; see Appendix A.

####  **GPS and Illuminance Measurement Systems and Scene-Camera** 

For measuring ambient illuminance of the environment, the X1 illuminance measurement head from Gigahertz-Optik was used. It was mounted to the right side of the driver’s headrest. Illuminance data are available in the dataset, but their evaluation will not be part of this paper. For collecting the GPS data, the Global Positioning System (GPS) was placed on the roof of the vehicle above the right rear passenger seat. The measurements were taken by a u-Blox EVK-M8 evaluation kit with a sampling frequency of 10 Hz. The Raspberry Pi read and stored both the GPS and illuminance data and synchronized both sensors. A Raspberry Pi HD camera was permanently installed to capture the environment outside of the vehicle.

The system’s calibration was achieved through the use of natural feature choreography, a method that involves the calibration and validation being conducted in real-world conditions that emulate the subject’s driving experience. This approach was essential to ensure the accuracy and precision of the calibration results.

#### 2.1.1. Calibration and Test Route

In the natural feature choreography, prominent or conspicuous points in the real world were used to calibrate the eye tracker. The test subject was told which point was to be fixated upon. The experimenter marked this point in the world camera image and the gaze vectors and target position were collected for calibration. Normally, 9 calibration points are sufficient. Other points should be selected for validation.

Only 8 pairs of markers are shown in the figure. The missing three are located on the left exterior mirror (pair 9), on the rear-view mirror (pair 10), and on the right side mirror (pair 11) of the vehicle used in order to maximize the viewing angle in the natural feature validation and calibration. The markers are placed relatively evenly in the relevant field of view. The furthest marker in the test is located at a distance of ≈57 m (marker pair 4). At this distance, the eyes are almost divergent ($\alpha =arctan(\frac{57}{63/2\;\cdot \;10^{-3}})=89.97{}^{\circ}$).

**Figure 7:**
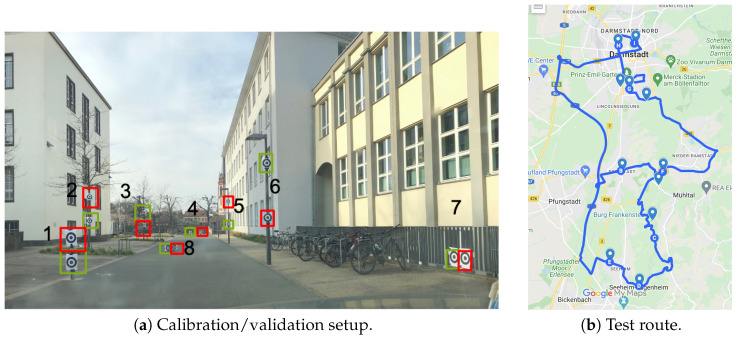
(**a**) The image shows the calibration setup during the day. The images were taken from the car and show the field of view of the suspended pairs of markers from 1–8; the markers used for validation are labeled in green and those used to calibrate the system in red. Panel (**b**) shows the test route driven by the subjects.

The route is around and within Darmstadt, Germany. The route is shown in Figure 7b. The track was deliberately chosen as an “8” so that almost equal numbers of left and right curves were passed. A strong overweighting of curves in one direction would shift the gaze distributions in the horizontal direction when the gaze distribution is considered over a large distance. The estimated route duration is 1 h and 34 min and the length ≈60 km.

#### 2.1.2. Daytime

The daytime trials started from 9:00 a.m. to 6:00 p.m., between March 2022 and December 2023 in Darmstadt. Depending on the season, sunset occurred between 5:00 p.m. and 9:30 p.m. The duration of the experiment was approximately 2 h 30 min.

#### 2.1.3. Nighttime

The night trial took place from 5 p.m. to 10 p.m., depending on the season. It was identical to the daytime trial.

### 2.2. Procedure

After adjusting the eye cameras of the head-mounted eye tracker to the subject, the experiment began with the calibration and validation of the head-mounted eye tracker using the natural feature choreography, but with the markers (subjects looked at the red marker to calibrate the system).

Before the start of the journey, the “Multidimensional Mood State Questionnaire” MDBF [27] was queried to assess the subject’s organismic factors. The test was then started. Halfway along the route, the questionnaire was asked again to record possible changes. The route was then continued. At the end, the natural feature validation was performed again to determine a potential slippage of the system on the head, and the MDBF was queried; see Appendix A. Before each test drive, the calibration of the world camera was checked and repeated if necessary.

In summary, the test procedure consists of the following steps:

1. Adjusting and setting up the eye tracking system to the test person ( 10 min).
2. Calibration to the natural feature choreography ( 5 min).
3. Validation on the natural feature choreography ( 15 min).
4. Questionnaire ( 5 min).
5. Driving Test route ( 45 min).
6 Questionnaire (half of the route) ( 5 min).
7. Continue route ( 45 min).
8. Questionnaire (end of the route) ( 5 min).
9. Validation on the natural feature choreography ( 15 min).

During the experiment, the experimenter sat in the passenger seat and navigated the subject. A natural atmosphere prevailed. In the following, the beginning to the middle of the route is referred to as route Section 1 and the middle to the end of the route as route Section 2.

#### Test Subjects

The test collective consisted of 20 test subjects (16 male, 4 female) aged between 18 and 54 years. Of the subjects, 7 had light-colored eyes and 13 had dark eyes (according to their own assessment).

According to their own statements, the subjects were not under the influence of drugs and did not wear glasses. Out of all subjects, 3 wore soft contact lenses.

### 2.3. Experiment Evaluation

Eye-tracking data were processed using Pupil Player software to detect blinks and fixations. Fixations were identified based on spatial and temporal thresholds; see Appendix B.1.1. Gaze points were filtered by pupil detection confidence, and blinks were excluded. Illuminance data were calibrated and interpolated to match gaze timestamps. GPS data were converted to longitude and latitude, then linked with environmental factors (e.g., street type, street name, and speed limits) using OpenStreetMap (OSM) data (same classification as Kobbert [8,24]; see Appendix B.2). The questionnaire data were averaged to assess participants’ mood, alertness, and calmness for the first half and the second half of the route; see Appendix A. The gaze accuracy and precision were calculated using interpolation based on validation data, generating an “accuracy map” which takes the angle depended accuracy into account; see Appendix B.4. The gaze data were processed and transformed into a car-centered coordinate system using homography; see Appendix B.1.1 and Appendix B.5. This allowed the integration of eye-tracking data with the scene camera image for further analysis.

Kernel density estimation (KDE) plots were utilized to visualize the distribution of gaze. Given the inability to ensure that test subjects received identical traveling times on distinct traffic sections, the test subjects of the data were weighted inversely to the number of data points per day/night per road environment, thus preventing individual subjects from being shown more or less in the plot. The calculation of the dispersion measures and fixation centroids (mean, median, standard deviation (std), and normalized median absolute deviation (nMAD)) of the gaze distribution was performed at the subject level, with the median of the metrics subsequently being taken.The nMAD is more robust to outliers as it is based on deviations from the median rather than the mean, and, unlike the standard deviation, it remains stable and unaffected by extreme values. The standard deviation assumes a symmetric (often normal) distribution, making it less suitable for skewed data.The nMAD relies on the median and performs better for asymmetrical distributions. When comparing variability across distributions with differing spread, the nMAD is advantageous due to its robustness and normalization, reducing distortions from heterogeneous or unevenly sized datasets, and preventing incorrect weighting of individual test subjects. The statistical analysis was performed using a Kruskal–Wallis test (due to the non-Gaussian distribution of the data and independence of the groups) followed by post hoc Conover tests with Holm correction to determine pairwise differences. If the group size was two, the test reduced to a Mann–Whitney U-test.

## 3. Results

The recorded eye-tracking data were recorded in the median with an accuracy and precision of 2.86°±0.11° during the day and 2.45°±0.04° at night. The lower accuracy during the day was due to the higher illuminance and direct sunlight and the associated reflections in the eye, or even direct illumination and overexposure of the eye cameras of the eye tracking system. This led to poorer detection of the pupil by the eye tracking system. Although the systems work in the infrared range, the sun also has a radiation amount in the infrared range. This source of interference is, of course, not present after sunset. The participants were in a good mood, wakeful, and rested during the test drives; see Appendix B.3. The results for day and night over the entire route are shown below. Then, the gaze distributions on the country road, highway, and in the city are compared with each other. Finally, two individual city roads are compared with each other.

### 3.1. Gaze Distribution Day vs. Night

When analyzing the gaze distribution over the entire route, it becomes apparent that the gaze distribution is wider during the day, both horizontally and vertically. This is also reflected in the dispersion measures of the standard deviation and the normalized median absolute deviation (nMAD), as shown in Table 2.

The standard deviation and nMAD of theta during the day are 15.05° and 8.66°, respectively, compared to 12.38° and 5.94° at night. When examining the vertical dispersion, the standard deviation and nMAD of phi during the day are 6.95° and 3.78°, respectively, compared to 6.19° and 2.67° at night. Significant reductions are observed in theta nMAD (p=0.0001) and phi nMAD (p=0.0102), indicating reduced variability in gaze angles during the night.

**Figure 8:**
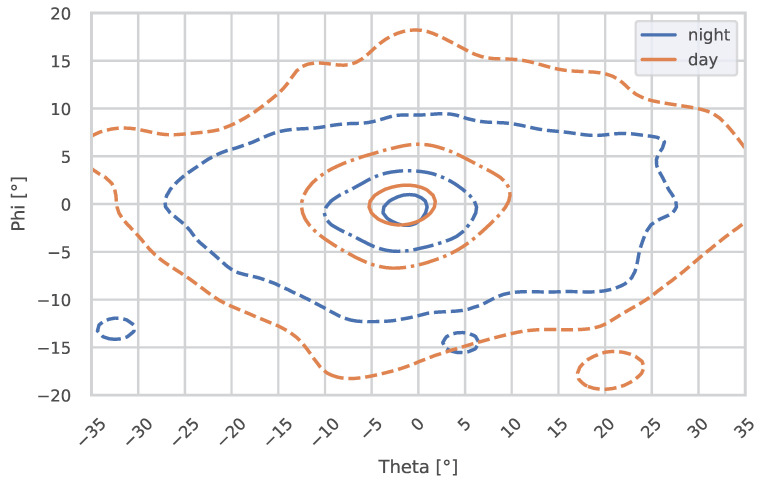
This figure shows the 10%, 50%, and 90% contours (from inside to outside) of the fixation distributions during the day and at night for the entire route. Theta represents the horizontal angle, and phi represents the vertical angle. The vanishing point of an infinitely straight road is located at 0° H/0° V.

The tendency for the gaze to be lowered at night can be seen in Figure 8 and in the data. With a median phi of 0.26° during daytime, the median at night is lower at −0.69°. Nevertheless, the analysis reveals no significant difference in theta and phi medians (*p* = 0.1763 and *p* = 0.9394, respectively).

### 3.2. Environment-Specific Comparisons

First, the inner environment differences between day and night are explored. An overview of the fixation centroid and the dispersion is given in the following Table 3.

**Figure 9:**
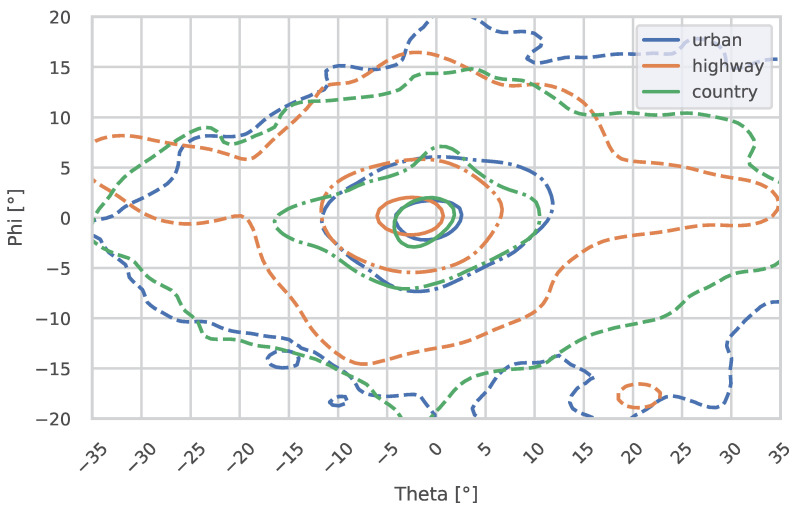
This figure shows the 10%, 50%, and 90% contours (from inner to outer) of the fixation distributions for urban areas, country roads, and highways during the **day**. Theta represents the horizontal angle, and phi represents the vertical angle. The vanishing point of an infinitely straight road is located at 0°/0°.

**Figure 10:**
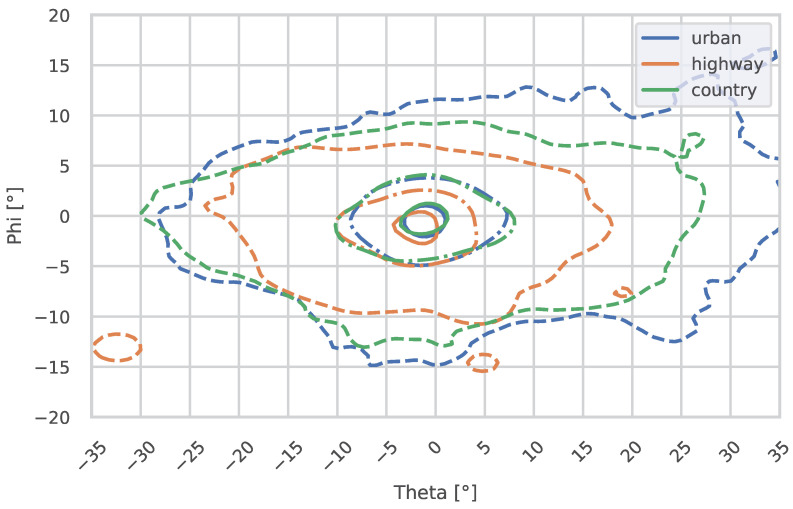
This figure shows the 10%, 50%, and 90% contours (from inner to outer) of the fixation distributions for urban areas, country roads, and highways during the **night**. Theta represents the horizontal angle, and phi represents the vertical angle. The vanishing point of an infinitely straight road is located at 0°/0°.

The following plots show the gaze distribution in urban areas, on country roads, and on highways, divided by daytime (Figure 9) and nighttime (Figure 10).

#### 3.2.1. Comparison of Day and Night Across Environments

The comparison between day and night across different environments—country, highway, and urban—revealed several insights regarding gaze behavior.

#####  **Country Roads** 

On country roads, the median of theta was slightly higher at night ($\Theta_{med\_day}=-0.73{}^{\circ}$, $\Theta_{med\_night}=-0.76{}^{\circ}$) but showed no significant differences (*p* = 0.9340). The nMAD of theta was significantly lower at night ($\Theta_{nMAD\_day}=9.00{}^{\circ}$, $\Theta_{nMAD\_night}=7.13{}^{\circ}$, *p* = 0.0022), indicating reduced variability in horizontal gaze. For the vertical focus point, the median of phi remained stable between day and night ($\Phi_{med\_day}=-0.42{}^{\circ}$, $\Phi_{med\_night}=-0.30{}^{\circ}$, *p* = 0.7281). The tendency of a lowered gaze could not be seen here. However, the nMAD of phi showed a significant reduction at night ($\Phi_{nMAD\_day}=3.58{}^{\circ}$, $\Phi_{nMAD\_night}=2.72{}^{\circ}$, *p* = 0.0451).

##### Highways

On highways, the theta metrics, including the median ($\Theta_{med\_day}=-2.66{}^{\circ}$, $\Theta_{med\_night}=-2.23{}^{\circ}$), nMAD ($\Theta_{nMAD\_day}=5.08{}^{\circ}$, $\Theta_{nMAD\_night}=5.08{}^{\circ}$), mean ($\Theta_{mean\_day}=-2.36{}^{\circ}$, $\Theta_{mean\_night}=-2.78{}^{\circ}$), and std ($\Theta_{std\_day}=8.86{}^{\circ}$, $\Theta_{std\_night}=8.36{}^{\circ}$), showed no significant differences (*p* > 0.05 for all comparisons).

For phi metrics, the median of phi tended to be lower at night compared to daytime and was close to significance ($\Phi_{med\_day}=0.38{}^{\circ}$, $\Phi_{med\_night}=-0.81{}^{\circ}$, *p* = 0.0564). Nevertheless comparing the mean of phi revealed significant differences ($\Phi_{mean\_day}=0.67{}^{\circ}$, $\Phi_{mean\_night}=-1.5{}^{\circ}$, *p* = 0.0264). The nMAD of phi was significantly lower at night ($\Phi_{nMAD\_day}=2.62{}^{\circ}$, $\Phi_{nMAD\_night}=1.67{}^{\circ}$, *p* = 0.0138).

#####  **Urban Roads** 

On urban roads, the horizontal gaze median showed a trend toward a lower value at night ($\Theta_{med\_day}=0.09{}^{\circ}$, $\Theta_{med\_night}=-0.22{}^{\circ}$, *p* = 0.0660). The nMAD of theta was significantly reduced at night ($\Theta_{nMAD\_day}=8.87{}^{\circ}$, $\Theta_{nMAD\_night}=6.11{}^{\circ}$, *p* = 0.0003). For the vertical gaze median ($\Phi_{med\_day}=0.08{}^{\circ}$, $\Phi_{med\_night}=-0.40{}^{\circ}$), no significant differences could be found, even though the trend of lowered gaze at night can be seen (*p* = 0.8912). However, the nMAD of phi was significantly lower at night ($\Phi_{nMAD\_day}=3.68{}^{\circ}$, $\Phi_{nMAD\_night}=2.98{}^{\circ}$, *p* = 0.0094). This is also visible in Figure 9 and Figure 10.

The comparison between day and night across different environments—country, highway, and urban—revealed several insights regarding gaze behavior.

#### 3.2.2. Differences in Gaze Distribution Between Environments

#####  **Nighttime Analysis** 

During the night, significant differences were observed in the theta medians across the three environments, as indicated by the Kruskal–Wallis test (p=0.0334). This suggests that the horizontal gaze distribution varies significantly depending on the road environment. However, the phi medians showed no significant differences between environments (p=0.3978), indicating that vertical gaze behavior remains consistent across road types at night, even though the lowered gaze on highways can also be seen in the plots; see Figure 10.

The post hoc Conover test for horizontal medians during the night revealed specific pairwise differences. A significant difference was observed between highway and urban environments (p=0.0315), suggesting distinct gaze behavior in these settings. The difference between country roads and highways showed a trend but was not statistically significant (p=0.1271). Similarly, the comparison between country roads and urban roads did not reveal significant differences (p=0.4519). For vertical medians, the post hoc test did not indicate any significant pairwise differences, with adjusted *p*-values all above 0.05 (e.g., highway vs. urban: p=0.6834; country vs. urban: p=0.9226).

#####  **Daytime Analysis** 

During the day, the Kruskal–Wallis test identified significant differences in the horizontal medians across the three environments (p=0.0020). In contrast, no significant differences were found in the vertical medians between environments (p=0.4089), suggesting that vertical gaze remains stable during the day.

The post hoc Conover test for horizontal medians during the day revealed significant pairwise differences. The comparison between highway and urban environments indicated significant differences in gaze behavior (p=0.0166). The difference between country and highway environments showed a trend but did not reach significance (p=0.0739). Similarly, the comparison between country and urban environments was not statistically significant (p=0.4534). For vertical medians, no significant pairwise differences were observed, consistent with the overall Kruskal–Wallis test result.

### 3.3. Residential vs. Mainroad

In this section, a main road and a residential road are compared. The Rheinstraße is a primary road featuring two to three lanes in each direction, separated by a tramway. The road is mostly straight and serves as a priority route.

In contrast, the Jahnstraße is a residential street with parking on both sides and bidirectional traffic, lacking central lane markings. The road is also mostly straight but includes numerous “right-before-left” priority situations and a small intersection with a roundabout.

At Rheinstraße in Darmstadt (Main Road), the theta median was −1.08°, with a theta nMAD of 5.13°. The phi median was −0.65°, and the phi nMAD was 2.23°. At Jahnstraße (Residential Road), the theta median was −1.43°, with a theta nMAD of 6.23°. The phi median was −1.25°, and the phi nMAD was 2.16°. Table 4 summarizes these results.

Looking at the horizontal dispersion, the gaze tends to be wider in the residential street. Especially when examining the 25% and 50% regions in the plots, this could be due to the parked cars on both sides of the street, where potential hazards can occur. Nevertheless, the theta nMAD did not show significant differences (*p* = 0.0575). Similarly, the horizontal medians also revealed no significant differences between the two streets (*p* = 0.7962).

For the vertical metrics, the gaze tends to be lowered in the residential street. This could be due to lower driving speeds or reduced street illumination. However, the phi metrics, including the vertical median (*p* = 0.5949) and vertical nMAD (*p* = 0.2807), were also not significantly different between the two streets. The ellipses and circles shown in the plots are based on the suggestions by Winter et al. [17], as discussed in Section 1.

## 4. Discussion

A comparison of the exact values of the metrics used across different studies is not recommended due to variations in the routes traveled, the types of eye-tracking devices employed, the eye-tracking accuracy, and the different data processing methods. Furthermore, the adjustment for the vanishing point was not made. Consequently, the authors of this paper investigated the common trends between the previous studies and the present one.

The findings align with previous research in several aspects. For instance, studies by Graf and Krebs (1976) [5] and Kobbert (2019) [8,24] consistently observed narrower gaze distributions at night compared to daytime, which was also evident in our results for all environments. The significant reductions in nMAD and std during the night further confirm this tendency. These reductions, particularly in urban areas and highways, reflect the limited field of view and the driver’s need to focus more intensely on the illuminated area, as highlighted by Brückmann and Diem [10,16].

In the highway environment, our results showed no significant differences in theta metrics between day and night, which is consistent with findings by Kobbert (2019). Nevertheless, when looking at phi, a significant difference was found for the mean (not median). When the 10% densest region is observed, in Figure 10, the the region of the highway is strongly lowered. The centroid of the fixations is also at −1.91° compared to 0.58° during the day. One potential explanation for this phenomenon is that the reduced gaze may be attributable to the constrained viewing distance. This is due to the absence of adaptive driving beam functionality in the test vehicle, which resulted in the low beam being activated for the majority of the time while driving on highways. This highlights the need for adaptive driving beams on highways. The relatively consistent and narrow gaze behavior on highways can be attributed to their standardized design, where drivers primarily focus on maintaining lane position and monitoring traffic. However, the significant reductions in phi nMAD at night indicate that vertical gaze behavior becomes more concentrated, likely due to reduced distractions and the driver’s focus on the road ahead. The potential for distractions could also serve as a contributing factor to the more pronounced horizontal gazes observed during daytime hours, which resemble the appearance of “Saturn” in the distribution.

Urban environments exhibited the most pronounced differences, with significant reductions in theta nMAD and std at night. These results align with Diem’s [16] findings that urban areas, due to their visual complexity, lead to broader gaze distributions during the day. Further, it can be seen that gaze in urban areas tends to be more focused on the right side when looking at theta values compared to the other environments; see Table 3. This could be due to the right-hand traffic and potential hazard near the driving lane. The significant reduction in dispersion at night suggests a shift toward a more focused gaze pattern, possibly driven by the need to navigate a more challenging visual environment in lower lighting conditions.

The comparison between Rheinstraße and Jahnstraße revealed no significant differences in the horizontal gaze dispersion metrics. Nevertheless it is close to significance. This is reflected in the larger horizontal nMAD, which can be seen at night (see Table 4). Also, the tendency for gaze to be lowered in the residential street is there, but no significance can be found.

**Figure 11:**
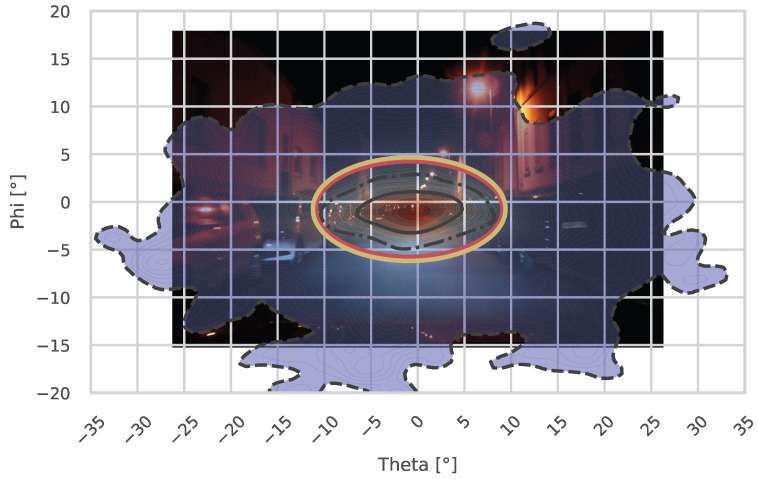
This figure shows the gaze distribution of the participants during the night across the Jahnstraße (residential road). The vanishing point of an infinitely straight road is located at 0° H/0° V. The three black contours represent the areas encompassing the 25%, 50%, and 95% most probable values of the kernel density estimation (*KDE*). Cooler colors indicate lower probabilities, while warmer colors represent higher probabilities. Additionally, the red contour shows the 20° H/10° V ellipse, and the yellow represents the 2· std° H/2 · std° V ellipse suggested by Winter et al. [17].

When comparing the proposed shapes from Winter et al. to fit the 95% contours of the fixations, it becomes evident that they do not align with the data observed in this study. Figure 11 illustrates the fixation distribution on Jahnstraße at night. The black lines indicate the 25% (solid), 50% (dashed-dotted), and 95% (dashed) probability density contours, as proposed by Winter et al. [17]. Additionally, the red contour shows the suggested 20° H/10° V ellipse, and the yellow contour represents the 2· std° H/2 · std° V ellipse. As the fixation distribution is not normally distributed, the proposed shapes do not adequately fit the 95% contour.

**Figure 12:**
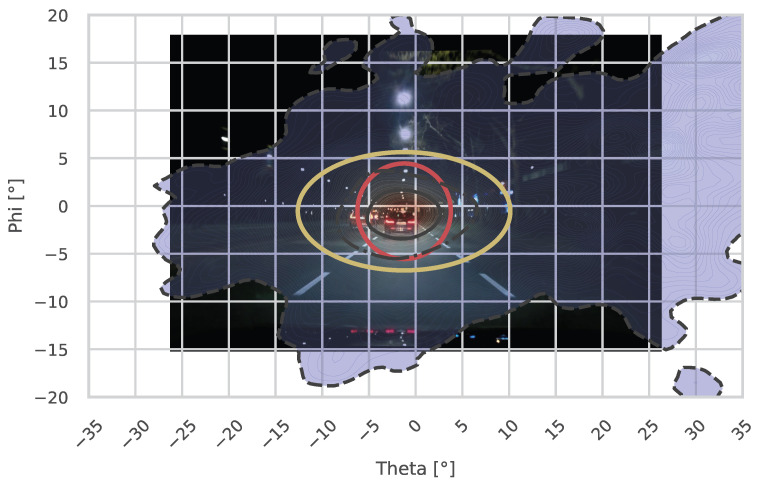
This figure shows the gaze distribution of the participants during the night across the Rheinstraße (main road). The vanishing point of an infinitely straight road is located at 0° H/0° V. The three black contours represent the areas encompassing the 25%, 50%, and 95% most probable values of the kernel density estimation (*KDE*). Cooler colors indicate lower probabilities, while warmer colors represent higher probabilities.Here, the red contour represents the suggested 10° circle, while the yellow contour illustrates the 2 · std° H/2 · std° V ellipse by Winter et al. [17].

When examining the main road (Rheinstraße), the results similarly fail to align with the proposed contours (see Figure 12). Here, the red contour represents the suggested 10° circle, while the yellow contour illustrates the 2 · std° H/2 · std° V ellipse.

The roads examined in this study share similar characteristics with the roads analyzed in Winter et al.’s research, yet the proposed fitting shapes fail to represent the fixation distributions observed here accurately.

The variability in gaze behavior and the methodological differences between studies pose significant challenges to using gaze distributions as a basis for designing lighting functions. As demonstrated in our data and supported by the literature [17], even slight changes in experimental setups or road conditions can lead to different gaze distributions. Additionally, the calibration and accuracy of eye-tracking systems remain critical issues [4,20], with errors potentially influencing the reliability of gaze measurements.

The findings of this study reveal that calculating a universal “holy grail” fixation point for each driving environment during the day or night is neither feasible nor meaningful. The gaze behavior of drivers varies significantly depending on multiple factors, as highlighted in the literature and confirmed by our results.

Given these challenges, the authors answer the posed question from the beginning: eye-tracking data are better suited for comparing lighting systems under controlled conditions rather than for designing them. Eye-tracking studies can provide valuable insights into how different lighting configurations influence gaze behavior and visibility. However, the goal should not be to direct the driver’s gaze but to ensure optimal illumination of all objects and areas within the visual scene, enabling drivers to assess and respond to hazards as needed.

Further drivers tend to scan the scene early for potential hazards, especially under good daylight conditions. In such scenarios, objects classified as non-threatening may no longer attract direct fixations, with drivers relying on peripheral vision instead. This behavior underscores the importance of providing optimal visibility across the entire scene rather than attempting to direct gaze to specific areas, without having direct scene context, like that proposed by Kobbert; see Figure 5 [8,24]. Our results support this observation, showing that gaze distributions during the day are broader and more variable, reflecting the driver’s ability to detect and assess hazards early.

The findings of this study highlight the need for adaptive lighting systems that dynamically respond to areas of poor visibility. High-definition headlamp systems, capable of selectively improving illumination in critical areas, offer a promising solution. By enhancing visibility where it is most needed, such systems can support drivers in maintaining situational awareness without constraining their natural gaze behavior.

Future lighting designs should prioritize ensuring good visibility across all objects in the driver’s field of view, allowing the gaze to follow naturally. This approach aligns with the principle that gaze behavior is not static but dynamically adapts to the driving context.

### Limitations and Concerns

Given that the participants were unevenly distributed (16 men and 4 women, aged 18–54), this may have influenced the calculated gaze distributions.

A recent study by Shen et al. showed that women tend to look more towards the information panel of the car and men tend to look more to the left of the horizon when driving on a straight road. The results are also questionable because only 10 subjects took part in the experiment, of which 4 were women and 6 were men. Of the 4 women, 3 were inexperienced, and for the men, only 2. Therefore, the inexperience of the women could also be a possible reason for this [28].

The effect of age does not seem to be negligible, as a meta-analysis by Ziv and Lidar in 2016 mentioned that “older drivers’ less effective visual scanning strategies are also associated with poorer road perception” [29]. Savage et al. also reported that “older drivers made fewer scans with a significant head movement component” [30]. However, in most of the studies mentioned, the older age group was, on average, 63 [31] or 67 years old [30].

Our subjects were mostly students with an age of 23.5±7.35 years (mean ± std). The data are, therefore, more representative of younger drivers. The effect of the 54-year-old participant does not fall into the typical “old” classification and is, therefore, negligible.

Our study focused on lighting conditions and road environments to generate generalized gaze distributions, without analyzing specific variable factors shown in Table 1. However, not considering these factors could have influenced the resulting gaze distributions. We assumed that potential influences would balance out over the total driving time of all subjects in the different environments. A more detailed event-based segmentation or semantic segmentation could allow a deeper analysis of these effects, but would require extensive data labeling and is better suited for a separate study.

## 5. Conclusions

In conclusion, this study demonstrates that there is no universal gaze distribution that can be used to design lighting functions. Gaze behavior is highly context-dependent and influenced by a multitude of dynamic and environmental factors. Eye-tracking remains a powerful tool for evaluating lighting systems in controlled conditions but should not serve as the sole basis for designing headlamp distributions. Instead, the focus should be on developing adaptive lighting systems that enhance visibility across the entire scene, allowing the gaze to follow naturally as drivers navigate complex environments.

## Figures and Tables

**Table 1:** This table describes the various factors in road traffic that can influence gaze behavior, according to Grüner et al. [1].

	Environmental Factors	Organismic Factors
*Non-variable*	Road geometry (curve, straight) lighting conditions (day/night, road lighting, headlights), road type (multi-lane, road surface), road environment (highway, city, country), weather conditions (e.g., dry, wet, rain, fog, snow).	Visual acuity [1,2], mental capacity [1,2], personality, driving experience [3], familiarity with the route [4].
*Variable*	Other road users (oncoming traffic preceding traffic, pedestrians, etc.), signs and displays (traffic lights, redirection signs, stop signs, etc.), objects and possible accidents, gaze and traffic (crossing, overtaking), interaction with the car (assistance systems), light-induced dynamics.	Cognitive load, exhaustion, alertness, drug influence.

**Table 2:** This table shows the mean, std, the median, and the nMAD of the horizontal (theta) and vertical (phi) gaze distribution. The data are grouped by day and night for a general comparison.

		Theta [°]				Phi [°]			
		Mean	Std	Median	nMAD	Mean	Std	Median	nMAD
Day		1.13	15.05	−0.31	8.66	0.02	6.95	0.26	3.78
Night		0.65	12.38	−0.39	5.94	−0.86	6.19	−0.69	2.67

**Table 3:** This table shows the mean, std, the median, and the nMAD of the horizontal (theta) and vertical (phi) gaze distribution. The data are grouped by road environment and time of day.

		Theta [°]				Phi [°]			
		Mean	Std	Median	nMAD	Mean	Std	Median	nMAD
Country	Day	−0.62	13.83	−0.73	9.00	−0.74	6.31	−0.42	3.58
	Night	−0.05	11.56	−0.76	7.13	−0.24	5.54	−0.30	2.72
Highway	Day	−2.36	8.86	−2.66	5.08	0.67	5.19	0.38	2.62
	Night	−2.78	8.36	−2.23	5.08	−1.50	4.20	−0.81	1.67
Urban	Day	2.06	15.47	0.09	8.87	−0.15	7.26	0.08	3.68
	Night	1.33	13.09	−0.22	6.11	−0.95	6.41	−0.40	2.98

**Table 4:** This table shows the descriptive statistics of horizontal (theta) and vertical (phi) gaze metrics at Rheinstraße and Jahnstraße during the night. Metrics include medians and normalized median absolute deviations (nMADs).

Street	Theta [°]		Phi [°]	
	Median	nMAD	Median	nMAD
Rheinstraße	−1.08	5.13	−0.65	2.23
Jahnstraße	−1.43	6.23	−1.25	2.16

**Figure A1:**
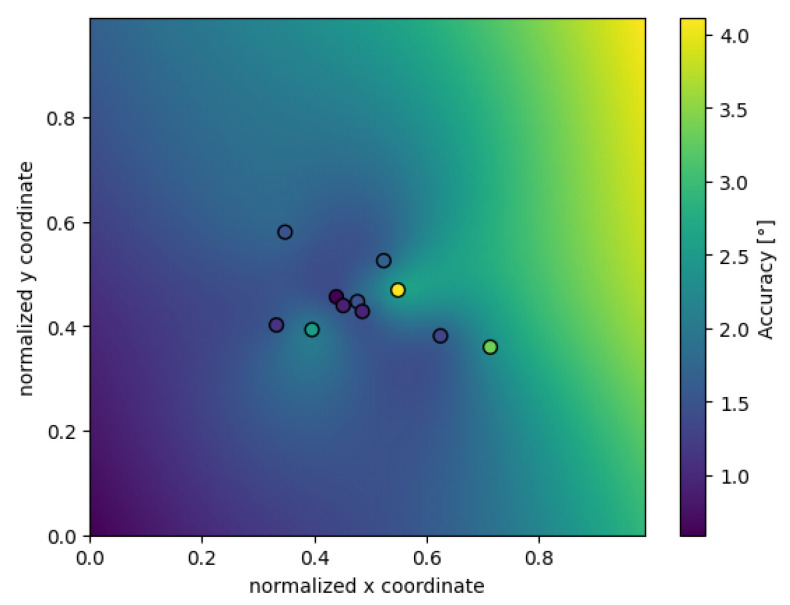
This figure shows a created accuracy map (median) for a participant. The x-axis and y-axis represent the normalized gaze position in the world coordinate image of the eye-tracking system. The marked points are the participant’s gaze angles when fixating on the markers. The color indicates the achieved accuracy.

**Figure A2:**
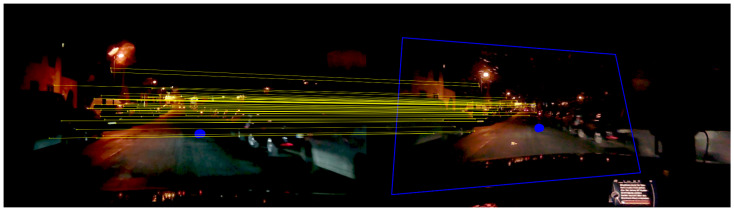
This figure illustrates the applied homography process. Gaze data from the right eye-tracking image are mapped to the corresponding coordinates in the left (scene camera) image.

## Data Availability

The raw data supporting the conclusions of this article will be made available by the authors on request.

## Abbreviations

The following abbreviations are used in this manuscript:

DFG	Deutsche Forschungsgemeinschaft (German Research Foundation)
GPS	Global Positioning System
LED	Light Emitting Diode
AFS	Adaptive Front Lighting System
KDE	Kernel Density Estimation
MDBF	Multidimensional Mood State Questionnaire
nMAD	Normalized Median Absolute Deviation
RBF	Radial Basis Function
API	Application Programming Interface
OSM	OpenStreetMap
Std	Standard Deviation
H	Horizontal
V	Vertical
RANSAC	Random Sample Consensus

## Appendix A. Questionaire

Steyer et al. define well-being as follows:

State of mind describes the momentary inner experience and feeling of an individual (“experientially represented”) and not observable behavior.

The MDBF - short form A by Steyer et al. is used to assess the variable organismic factors or the state of mind of the test subjects [27]. The dimensions are divided into good–bad mood, alertness–fatigue, and rest–restlessness. The dimensions contain the following items:

**good–bad mood**	**wakefulness–fatigue**	**rest–restless**
- satisfied - bad - good - unwell	- rested - tired - sleepy - lively	- restless - calm - restless - relaxed

The items in the respective dimension are formulated as a statement, e.g., “I am satisfied”. The respondent rates the statement using a 5-point Likert scale, where 1 means “disagree”, 2 means “disagree somewhat”, 3 means “somewhat agree”, 4 means “agree somewhat”, and 5 means “agree”. For the analysis, the Likert values of the items that assess bad mood, tiredness, and restlessness are inverted and then the mean value is calculated for the dimensions. Likert values greater than 3 indicate good mood, alertness, and calmness in the respective dimension.

## Appendix B. Experiment Evaluation

The experimental setup, using the available measurement instruments, including an eye-tracking system, GPS, illuminance sensor, static scene camera (Raspberry Pi HQ), and questionnaires, allows for the determination of influences on gaze behavior. However, extensive post-processing of the data is required. This section describes how the eye-tracking data are processed and how the data from other measurement instruments are assigned to each data point.

The following describes how the eye-tracking data are processed.

### Appendix B.1. Eye-Tracking Data

The eye-tracking data for each participant must be loaded into the post-processing software “Pupil Player” to be converted into common file formats (such as .csv). Additionally, in this step, blinks and fixations of the participants are detected.

#### Appendix B.1.1. Fixations

In the Pupil Player software, fixations are detected using a dispersion-based algorithm [25]. If consecutive gaze points are within a certain threshold (measured in degrees, spatially) from each other within a specified time window, they are recognized as a fixation. Pupil Player attempts to maximize the fixation duration within the time window. If the time window spans 100 to 600 ms and the software detects two consecutive 300 ms fixations, they are merged into a single fixation. The following parameters need to be set [32]:

- Maximum dispersion (spatial, degrees): The maximum distance between all gaze points during a fixation.
- Minimum duration (temporal, ms): The minimum duration during which the dispersion threshold must not be exceeded.
- Maximum duration (temporal, ms): The maximum duration during which the dispersion threshold must not be exceeded [32].

The spatial dispersion threshold is set to 2°, the minimum duration to 100 ms, and the maximum duration to 600 ms. Due to the dynamic environment during the experiment, the maximum dispersion was chosen to be larger, as the objects being fixated on move relative to the driver’s position.

The gaze data are associated with pupil data (diameter (mm, px)), blink information, and fixation data. The software assigns each data point a confidence value indicating how reliably the pupil was detected. Here, a value of 0 indicates poor detection and a value of 1 indicates good detection. Data points recorded during blinks or with a confidence lower than 0.6 are filtered out.

### Appendix B.2. GPS

The GPS data are converted into longitude and latitude and then associated with the non-variable environmental factors using the API of the OSM database [33]. This allows each GPS point (longitude and latitude) to be associated with street names, surroundings (city, countryside, highway), speed limits, the number of lanes, and current speed. Each gaze point was assigned the information that was temporally closest. The division into city and countryside was carried out according to the same categorization as in Kobbert’s work (2019) [8].

The roads were categorized as follows:

City streets:

- All roads within the traversed cities or towns.
- Connecting roads between cities or towns with a speed limit ≤ 50 km/h.

Country roads:

- Roads connecting cities or towns with a speed limit > 50 km/h and two lanes.
- Roads connecting cities or towns that are single-lane with a maximum permitted speed ≤ 100 km/h.

For the highway, only the “A5” highway was considered, as this was the only highway driven during the experiment. A detailed description of road categorization according to OSM data can be found in Kobbert’s work; see [8].

### Appendix B.3. Questionnaire

The dimensions of the MDBF were determined for Section 1 by averaging the data collected before and in the middle of the experiment (T1–T2). For Section 2, the data were averaged in the middle and at the end (T2–T3).

Each participant was assigned as to whether they were in a good or bad mood, awake or tired, and calm or restless for the two sections.

The results are shown in the following table.

**Table A1:** Comparison of MDBF Likert Values Across Moments.

Moment	Good–Bad Mood	Rest–Restless	Wakefulness–Fatigue
Likert value T1–T2	4.739645	4.559524	3.976190
Likert value T2–T3	4.755952	4.589286	3.797619

### Appendix B.4. Accuracy and Precision Map

During the Natural Feature Choreographie, the gaze angles for each marker during validation, as well as the determined accuracy (median) and precision (median), were stored.

This allows accuracy and precision for all gaze angles to be estimated before and after the experiment based on the 11 resulting pairs. The 11 determined pairs are assumed to be “true” values, and accuracy for all other gaze angles is interpolated. An RBF interpolation with a “Thin-Splate-Spline” was chosen. A resulting accuracy map is shown in Figure A1. As the gaze vector is mapped onto the world camera image of the head-mounted eye tracker, the resulting x- and y-coordinates in the image reflect the participant’s gaze angles.

To compensate for the effect of slippage, the determined functions were averaged before and after the experiment.

### Appendix B.5. Representation of Data in the Scene Camera Image

The previous section describes how the eye-tracking data were associated with the data from other measurement instruments. However, the eye-tracking data are displayed concerning the world camera image and not the scene camera image. The following section describes how these are displayed in the scene camera image and explains how the gaze distributions are presented by visual angle.

#### Transformation of Gaze Data into a Car-Centered Coordinate System

The most complex part of post-processing is transforming the head-centered gaze data into car-centered data. This is achieved by calculating a homography for each image pair in the recorded videos. As the recordings of the world camera of the eye-tracking system and the scene camera are both set to a frame rate of 30 fps, there should be a corresponding frame in the other video for each frame in the videos. However, it turned out that the eye-tracking software had a frame rate of ∼29 fps during the day and ∼17 fps at night. The drop at night could have occurred due to the longer exposure time required.

Therefore, the videos must first be resampled to the same frame rate. The scene camera video was adjusted to the frame rate of the world camera as the eye-tracking software’s data, such as the gaze vectors, are associated with a specific frame number.

Next, the distortion of the corresponding images is corrected using camera calibration. The gaze data associated with the image are also undistorted. After undistortion, it is assumed that straight lines in the real world are now represented as such in the image and that the conditions for calculating the homography are met. Next, the world camera image is enlarged to 1920 × 1080 px.

During night drives, the images are gamma-adjusted to brighten them. Then, the world camera image is color-matched to the scene camera image using a histogram match.

Subsequently, interest point matching is performed for the image pair. The steps of detection, description, and matching are performed using the neural network “SuperGlue” [34]. The homography can then be calculated using a graph-cut-based RANSAC algorithm [35].

With the obtained homography, the gaze point from the world camera image of the eye-tracking glasses can be transformed onto the scene camera. This procedure is repeated for each image pair. An image sequence for the individual steps is shown in Figure A2.

During the day, a homography could be calculated in 82.46% of corresponding frames, whereas at night, this value was 57.38%.

## References

[1] Grüner M., Ansorge U. (2017). Mobile Eye Tracking During Real-World Night Driving: A Selective Review of Findings and Recommendations for Future Research. J. Eye Mov. Res..

[2] Sun Q.C., Xia J.C., He J., Foster J., Falkmer T., Lee H. (2018). Towards unpacking older drivers’ visual-motor coordination: A gaze-based integrated driving assessment. Accid. Anal. Prev..

[3] Mourant R.R., Rockwell T.H. (1972). Strategies of Visual Search by Novice and Experienced Drivers. Hum. Factors: J. Hum. Factors Ergon. Soc..

[4] Mourant R.R., Rockwell T.H. (1970). Mapping eye-movement patterns to the visual scene in driving: An exploratory study. Hum. Factors.

[5] Graf C.P., Krebs M.J. (1976). Headlight Factors and Nighttime Vision: Final Report.

[6] Rockwell T.H., Ernst R.L., Rulon M.J. (1970). Visual Requirements in Night Driving.

[7] Zwahlen H.T. (1982). Driver Eye Scanning on Curves and on Straight Sections on country Highways. Proc. Hum. Factors Soc. Annu. Meet..

[8] Kobbert J. (2019). Optimization of Automotive Light Distributions for Different Real Life Traffic Situations. Ph.D. Thesis.

[9] Brimley B.K., Carlson P.J., Hawkins H.G. (2014). Use of Fixation Heat Maps to Evaluate Visual Behavior of Unfamiliar Drivers on Horizontal Curves. Transp. Res. Rec. J. Transp. Res. Board.

[10] Brueckmann R., Chmielarz M., Churn J., Gottlieb W., Hatzius J., Hosemann A., Reitter C., Roessger P., Schneider W., Sprenger A. (2000). Blickfixationen und Blickbewegungen des Fahrzeugführers sowie Hauptsichtbereiche an der Windschutzscheibe. Fat-Schriftenreihe Fat.

[11] Cengiz C., Kotkanen H., Puolakka M., Lappi O., Lehtonen E., Halonen L., Summala H. (2014). Combined eye-tracking and luminance measurements while driving on a rural road: Towards determining mesopic adaptation luminance. Light. Res. Technol..

[12] Mortimer R.G., Jorgeson C.M. (1974). Eye Fixations of Drivers in Night Driving with Three Headlight Beams.

[13] Damasky J., Hosemann A. (1998). The Influence of the Light Distribution of Headlamps on Drivers Fixation Behaviour at Nighttime.

[14] Huhn W. (1999). Anforderungen an Eine Adaptive Lichtverteilung für Kraftfahrzeugscheinwerfer im Rahmen der ECE-Regelungen.

[15] Damasky J. (1995). Lichttechnische Entwicklung von Anforderungen an Kraftfahrzeug-Scheinwerfer. Ph.D. Thesis.

[16] Diem C. (2005). Blickverhalten von Kraftfahrern im Dynamischen Straßenverkehr. Ph.D. Thesis.

[17] Winter J., Fotios S., Völker S. (2017). Gaze direction when driving after dark on main and residential roads: Where is the dominant location?. Light. Res. Technol..

[18] Shinar D., McDowell E.D., Rockwell T.H. (1977). Eye movements in curve negotiation. Hum. Factors.

[19] Land M.F., Lee D.N. (1994). Where we look when we steer. Nature.

[20] Schulz R. (2012). Blickverhalten und Orientierung von Kraftfahrern auf Landstraßen. Ph.D. Thesis.

[21] Hristov B. (2010). Untersuchung des Blickverhaltens von Kraftfahrern auf Autobahnen. Ph.D. Thesis.

[22] Kobbert J., Erkan A., Bullough J., Khanh T. (2023). A Novel Way of Optimizing Headlight Distributions Based on Real-Life Traffic and Eye Tracking Data Part 3: Driver Gaze Behaviour on Real Roads and Optimized Light Distribution. Appl. Sci..

[23] Kobbert J., Erkan A., Bullough J., Khanh T. (2023). A Novel Way of Optimizing Headlight Distributions Based on Real Life Traffic and Eye Tracking Data Part 1: Idealized Baseline Distribution. Appl. Sci..

[24] Kobbert J., Erkan A., Bullough J., Khanh T. (2023). A Novel Way of Optimizing Headlight Distributions Based on Real-Life Traffic and Eye-Tracking Data Part 2: Analysis of Real-World Traffic Environments Data in Germany. Appl. Sci..

[25] Pupil Labs Core—Best Practices—Pupil Labs. https://docs.pupil-labs.com/core/best-practices/.

[26] Kassner M., Patera W., Bulling A. Pupil: An Open Source Platform for Pervasive Eye Tracking and Mobile Gaze-based Interaction. Proceedings of the Adjunct Proceedings of the 2014 ACM International Joint Conference on Pervasive and Ubiquitous Computing.

[27] Steyer R., Schwenkmezger P., Notz P., Eid M. (2004). Entwicklung des Mehrdimensionalen Befindlichkeitsfragebogens (MDBF), Primärdatensatz, (Version 1.0.0) [Data and Documentation].

[28] Shen N., Zhu Y., Oroni C.Z., Wang L. Analysis of Drivers’ Eye Movements with Different Experience and Genders in Straight-line Driving. Proceedings of the 2021 IEEE International Conference on Dependable, Autonomic and Secure Computing (DASC), International Conference on Pervasive Intelligence and Computing (PiCom), International Conference on Cloud and Big Data Computing (CBDCom), and International Conference on Cyber Science and Technology Congress (CyberSciTech).

[29] Ziv G., Lidor R. (2016). The Effect of Age on Gaze Behavior in Older Drivers and Pedestrians—A Review. J. Eye Mov. Res..

[30] Savage S.W., Zhang L., Swan G., Bowers A.R. (2020). The Effects of Age on the Contributions of Head and Eye Movements to Scanning Behavior at Intersections. Transp. Res. Part F Traffic Psychol. Behav..

[31] Reimer B., Mehler B., Wang Y., Coughlin J.F. (2010). The Impact of Systematic Variation of Cognitive Demand on Drivers’ Visual Attention across Multiple Age Groups. Proc. Hum. Factors Ergon. Soc. Annu. Meet..

[32] Pupil Labs Core—Terminology—Pupil Labs, 6/15/2022. https://docs.pupil-labs.com/core/terminology/.

[33] OpenStreetMap. https://www.openstreetmap.org/about.

[34] Sarlin P.E., DeTone D., Malisiewicz T., Rabinovich A. SuperGlue: Learning Feature Matching with Graph Neural Networks. Proceedings of the IEEE/CVF Conference on Computer Vision and Pattern Recognition.

[35] Barath D., Matas J., Noskova J. MAGSAC: Marginalizing Sample Consensus. Proceedings of the 2019 IEEE/CVF Conference on Computer Vision and Pattern Recognition (CVPR).

